# Immune checkpoint inhibitor in recurrent hypermutated glioblastoma with *POLE* mutation

**DOI:** 10.1093/noajnl/vdab093

**Published:** 2021-06-28

**Authors:** Sith Sathornsumetee, Sarun Nunta-aree, Pornsuk Cheunsuchon

**Affiliations:** 1 Department of Medicine (Neurology), Faculty of Medicine Siriraj Hospital, Mahidol University, Bangkok 10700, Thailand; 2 Department of Surgery (Neurosurgery), Faculty of Medicine Siriraj Hospital, Mahidol University, Bangkok 10700, Thailand; 3 Department of Pathology, Faculty of Medicine Siriraj Hospital, Mahidol University, Bangkok 10700, Thailand

**Pathogenic mutation of DNA polymerase epsilon catalytic subunit (*POLE*) is associated with high tumor mutation burden (TMB) and response to immune checkpoint inhibitor (ICI) in several cancers. Owing to its rarity, clinical trial to evaluate the ICI efficacy in *POLE*-mutated glioblastoma is very challenging. Here, we report a case of recurrent *POLE-*mutated glioblastoma with response to pembrolizumab and bevacizumab. With growing real-world evidence, we propose that ICI may be considered among the treatment options for recurrent hypermutated glioblastoma with *POLE* mutation.**

**Figure 1.**
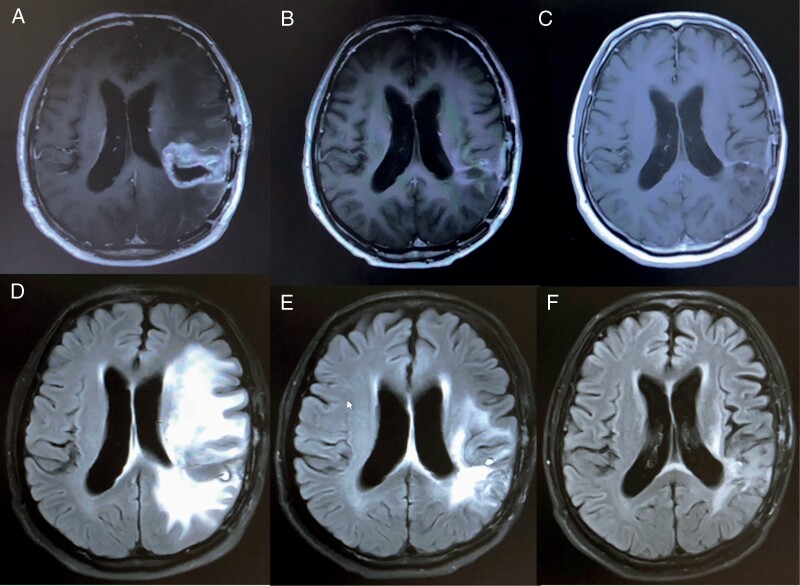
Brain MRI demonstrated recurrent *POLE*-mutated glioblastoma with partial response to pembrolizumab and bevacizumab. T1W + Gadolinium sequence in the upper panel (A-C) and T2W/FLAIR sequence in the lower panel (D-F). (A) and (D) at baseline; (B) and (E) at 3 months after pembrolizumab plus bevacizumab; and (C) and (F) at 12 months after pembrolizumab plus bevacizumab. T1W, T1-weighted; T2W, T2-weighted; FLAIR, Fluid-attenuated inversion recovery.

A 65-year-old man presented with naming difficulty and confusion in October 2016, and brain MRI revealed left parietal enhancing tumor. He underwent awake craniotomy with gross total resection of tumor in November 2016. Pathology confirmed glioblastoma (WHO grade IV), *IDH* wild type. Standard radiotherapy with concurrent temozolomide followed by adjuvant temozolomide was administered. MRI after 3 cycles of temozolomide demonstrated progression that prompted a repeat resection with pathology confirming recurrent glioblastoma. Tumor multigene panel (FoundationOne) revealed microsatellite stable, high TMB of 32 mutations/Mb. Genomic alterations were identified with mutations of *NF1* E524, *PIK3CA* G118D, *PTEN* D24Y, E299, *CBL* splice site 1096-1 G>T, *CHD2* E1542, *CSF1R* F971fs*7, *PIK3R1* R348, *POLE* P286R, *SETD2* E463, E581 and *TP53* P151S, and R213. These mutations were determined as clonal events. The pathogenic *POLE* P286R is the most common somatic mutations involving the proofreading domain leading to hypermutation with neoantigen abundance and T-cell infiltration rendering response to ICI in several cancers.^1,2^ One month after resection, tumor progression was noted. Therefore, pembrolizumab 200 mg with bevacizumab 10 mg/kg were given every 3 weeks with neurologic improvement and without significant side effects. Our reason to combine bevacizumab was for symptom control from edema requiring corticosteroids (Figure 1A and D). As corticosteroids may impede ICI efficacy, bevacizumab may serve as an alternative agent. In addition, combinations of immunotherapy and antiangiogenic therapy have shown promise in preclinical studies with more than 90 clinical trials evaluating various combinations in several cancers.^3^ Follow-up MRIs at 3, 6, and 12 months confirmed partial response (Figure 1). Pembrolizumab and bevacizumab were continued for 15 months with sustained clinical and radiographic improvement. However, MRI demonstrated progressive disease at 15 months. A combination of anti-cytotoxic T-lymphocyte antigen 4 (CTLA-4), ipilimumab, another anti-PD1, nivolumab, and bevacizumab was administered with no response. The patient went on palliative care with the overall survival from the start of bevacizumab plus pembrolizumab of ~20 months. 

It is challenging to attribute the response observed in our patient to pembrolizumab, bevacizumab, or both. Prior case reports demonstrated response to ICI monotherapy (pembrolizumab or atezolizumab) in *POLE*-mutated glioblastoma.^4,5^ Although there is no specific study of bevacizumab monotherapy in *POLE*-mutated glioblastoma, the duration of response in our patient (15 months) was longer than that typically seen with bevacizumab monotherapy (~4 months from the BRAIN study^6^) in recurrent unselected glioblastoma. The length of response for more than 1 year may argue against the sole effect of bevacizumab alone on response in our patient. A recent randomized phase II study failed to demonstrate the efficacy of either pembrolizumab alone or pembrolizumab plus bevacizumab in recurrent glioblastoma.^7^ However, TMB and mutational profiles including *POLE* mutation were not reported in this study. At present, there is only one prior report of recurrent *POLE*-mutated glioblastoma displaying a protracted response to pembrolizumab and bevacizumab.^8^ Taken together, the prolonged response seen in our patients was more likely due to either pembrolizumab or pembrolizumab plus bevacizumab.

Two recent analyses of various cancers demonstrated that high TMB was associated with ICI benefit in cancers related to environmental carcinogens, such as lung cancer, melanoma, bladder cancer.^1,9^ Another high TMB subset associated with ICI response was pan-cancers with either mismatch repair deficiency or polymerase deficiency from pathogenic mutation of *POLE* or *POLD1*.^1,2^ Our case here along with prior reports^4,5,8^ timely confirmed that this observation also extended to include glioblastoma and may support the use of ICI in hypermutated glioblastoma with *POLE* mutation. In contrast, ICI should not be routinely used in glioblastoma with high TMB from a more common setting of temozolomide exposure that is less likely respond to ICI, particularly amidst the controversial FDA approval of pembrolizumab in refractory pan-cancers with high TMB.^10^ Although our patient had a protracted response to ICI, resistance eventually developed. More understanding of genomic heterogeneity and immunologic mechanisms underlying acquired resistance to ICI is needed. While waiting for more trial results, current real-world evidence, albeit limited due to its rarity, may suggest that ICI can be considered among salvage therapies for *POLE*-mutated recurrent glioblastoma.
